# Food for love: the role of food offering in empathic emotion regulation

**DOI:** 10.3389/fpsyg.2014.00032

**Published:** 2014-01-31

**Authors:** Myrte E. Hamburg, Catrin Finkenauer, Carlo Schuengel

**Affiliations:** Department of Clinical Child and Family Studies, VU University AmsterdamAmsterdam, Netherlands

**Keywords:** food offering, interpersonal processes, emotion regulation, empathic concern, eating

## Abstract

The present article examines the interpersonal and intrapersonal antecedents and consequences of food offering. Food offering is one of the earliest biobehavioral regulatory interactions between parent and child. It ensures survival of the child who is fully dependent on food provision by others. The quality of these early interactions influences how people respond to situations later in life, and food offering in particular may be closely related to emotion regulation throughout the lifespan. While research has examined other forms of emotion regulation, and food consumption has been studied from an *intra*personal perspective, we know little about the *inter*personal effects of food offering. After reviewing literature from a wide range of disciplines, we propose that one mechanism underlying these effects is empathic emotion regulation (EER). We conceptualize EER as an interpersonal regulation system in which an empathic response to another person’s emotional state aims to regulate both emotion within the provider and across interaction partners. We suggest that the offer of food by an empathic provider is motivated by the emotional state of one’s interaction partner (recipient). By offering food, the provider not only aims to attenuate the recipient’s negative affect but also her own. Food offering thereby becomes a means to increase positive affect for both recipient and – when the offer has the desired effect – provider. We further propose that the sharing of food resources as well as the use of food as a support behavior increases interpersonal closeness. Finally, we frame the process of food offering within a developmental perspective. If the regulatory success of food offering becomes a replacement for other support behaviors, children will learn from an early age to use food as a primary means to soothe self and others, possibly resulting in eating disorders and a restricted range of coping behavior.

My doctor told me to stop having intimate dinners for four unless there are three other people.

–Orson Welles

(in Giriodi, 2010. Breakfast in Paris, Lunch in Rome, Dinner in London, p. 233)

Food is a fundamental human need that influences both physiological and emotional states. As such, the search for and consumption of food has shaped human and animal behavior. People feel strongly about their individual food preferences and the food culture they were raised in. Eating behavior goes beyond nutrition and alleviating hunger; family, friends, and cultural heritage shape individual food preferences. Food offering can be used to show affection to loved ones, to show hospitality to strangers, or to adhere to or express religious beliefs. The present article explores the interpersonal properties of food offering, investigating the emotional component involved in the offer and reception of food. As illustrated by the Orson Welles’ epigraph, we urge food researchers not to overlook the importance of other people at the dinner table.

Infants learn from an early age to associate food with soothing and social interaction (Smith et al., 1990; Moens et al., 2007; Stifter et al., 2011). The physiological properties of food affect mood by way of neurotransmitters (Markus et al., 1998) and endocrine responses (Dallman et al., 2003). Intake of food items has been shown to decrease feelings of helplessness, depression, loss of control, and distress (Markus et al., 1998), decrease stress (Oliver et al., 2000) and increase feelings of joy (Macht and Dettmer, 2006). Above and beyond physiological effects, food has the capacity to enhance positive affect by way of association with situations or contexts (Locher et al., 2005). Food items do not merely represent a means to satiety, but can also signify comfort or reward. For example, opening a bottle of champagne often signals a celebration of success, and eating (lots of) ice cream often signifies consolation after a disappointment. To date, using food to regulate emotions has been studied primarily from an *intra*personal perspective, examining emotional effects within the individual. Nevertheless, the possibility that people may experience emotional effects due to the *inter*personal regulatory processes related to food offering certainly merits closer investigation.

The food infants and young children gain access to depends mostly, if not solely, on what others offer them. Later in life, people prepare and offer food to friends, acquaintances, romantic partners, children, and sometimes even strangers. The food items offered may vary as a function of the expression of emotion by others and often are a metaphor for comfort, reward, or celebration (Locher et al., 2005). In this article, we suggest that food offering plays an important role in what we call *empathic emotion regulation* (EER). We suggest that the offer of food is motivated by – and results in the regulation of – the emotional state of both provider and receiver. We further propose that offering food resources as well as the use of food as a support behavior increases interpersonal closeness.

In the sections that follow, we review the literature and introduce a new conceptual model that could guide future research. In the section “Food and Emotion Regulation,” we review literature on food and emotion regulation from a wide range of disciplines. In “Social Aspects of Eating” and “Regulating Emotions with Comfort Food,” we argue that eating behavior and interpersonal processes are inextricably intertwined and, in “Empathic Emotion Regulation,” we propose that EER stands at the root of this connection. In “EER Through Food Offering,” we set forth our views of how EER through food offering can decrease negative affect, increase positive affect and increase interpersonal closeness. Finally, we propose that EER through food offering can have both functional and dysfunctional consequences and suggest in “Future Directions,” that incorporating the interpersonal functions of food in future research will facilitate a better understanding of the development of disordered eating.

## FOOD AND EMOTION REGULATION

The motivation to eat is not merely driven by a desire for nutrients and satiety; emotional, and psychological processes play an important role as well. Emotional states affect when people eat, how much they eat, and which food items they choose to consume. Consuming food, in turn, affects subsequent emotional states (Macht, 2008). Even in 1-day-old infants sucrose solutions provide a calming effect (Smith et al., 1990). People change their eating patterns as a response to negative emotions (Greeno and Wing, 1994). Researchers showed, for instance, that when daily hassles increased, women with high cortisol reactivity increased their food intake (Newman et al., 2007). People who are stressed report eating more high-energy, snack-type food (Oliver and Wardle, 1999; Oliver et al., 2000), and people presented with unsolvable anagrams eat more chocolate and fewer grapes than people presented with solvable anagrams (Zellner et al., 2006). Induction of a depressive mood state increased chocolate craving (Willner et al., 1998) and intake of sweet foods (Lowe and Maycock, 1988). Michels et al. (2012) found that children respond to problems by consuming more sweet and fatty food and less fruit and vegetables.

Research suggests that eating – or choosing certain food items over others – can indeed attenuate negative psychological states. Markus et al. (1998) showed that a rich-carbohydrate, low-protein diet decreased feelings of helplessness, depression, loss of control, and distress by raising the level of serotonin in the body. Dallman et al. (2003) suggested that food consumption can act as a form of self-medication, where stress-induced glucocorticoids increase motivation for fat and insulin. The consumption of fat and insulin, in turn, leads to reduced activity of the hypothalamic-pituitary-adrenal axis (HPA axis, controls the neuroendocrine response to stress).

Even in the absence of direct stressors, many foods appear to have positive effects on mood. Macht and Dettmer (2006) asked participants to record their mood state twice daily for a week after consuming an apple, a bar of chocolate, or no food at all. Although the researchers found equal levels of satiation after consumption of either the apple or chocolate, participants reported more joy and elevated mood after eating chocolate. Although less so than chocolate, the apple also elevated participants’ mood when compared with participants in the no food control condition. Because sweet food has been shown to reduce stress and sensitivity to pain (Smith et al., 1990; Harrison et al., 2012), Smith et al. (1990) proposed that the opioid pathways responsible for the effects of morphine may also explain the analgesic effect of sugar in young infants. Consequently, food items become associated with uplifting effects (Schellekens et al., 2012).

## SOCIAL ASPECTS OF EATING

Food preferences are not shaped in isolation; eating is an inherently social behavior. A meal shared with others is held in higher esteem and regarded as more of a proper meal than food consumed by oneself (Sobal et al., 2002). Infants are fully dependent upon caregivers for food provision and become conditioned to associate having their needs met with the presence of others (Hofer, 2006). Kin selection (Hamilton, 1964) provides one explanation for why people are willing to forfeit food for the sake of feeding family members. Nevertheless, food sharing appears to be a highly adaptive trait even among non-family members in that it may facilitate cooperation, allow for relationship maintenance, and create mating opportunities (Jaeggi and Van Schaik, 2011). Thus, the costs of sharing food resources with others, including strangers, are outweighed by the social benefits that food offering provides.

Just how much the presence of others influences eating behavior is highlighted by a diary study showing that the closer the relationship with someone, the larger the meal people ate in the presence of that person (De Castro, 1994). People tended to have larger meals when eating with, for example, family members and close friends than when eating with colleagues or classmates. Meal size decreased as social intimacy decreased, with meals being smallest when consumed alone. In line with these results, Koh and Pliner (2009) found that participants in the lab consumed more pasta when eating with a friend than with a stranger. Distraction and increased meal duration cannot entirely account for this social facilitation effect (Hetherington et al., 2006; Brindal et al., 2011), indicating that the presence of others in and of itself is an important determinant of (increased) food intake. Hermans et al. (2012) found that participants mimic the person they are eating with, preferring to take a bite when their partner did instead of eating at their own pace. Howland et al. (2012) showed that having been exposed to the eating behavior of friends influenced subsequent eating behavior even when alone, indicating that the eating behavior of others may signal a social norm (e.g., eating small portions) strong enough to carry over into non-social settings.

Research has shown that social relationships not only influence eating behavior, but that eating behavior can also be a reflection of – or even serve to strengthen – relationships. For example, when two people offered one another food, observers rated their relationship as closer than when no food was offered. If two people shared food by feeding one another observers rated their relationship as even closer (Miller et al., 1998; Erwin et al., 2002). Based on their findings, Miller et al. (1998) concluded that food sharing and feeding are important non-verbal indicators of friendship and romantic involvement. Alley (2012) and Alley et al. (2013) replicated these results and, additionally, found that participants perceived people sharing food as more attracted to one another. Consistent with these findings, Kniffin and Wansink (2012) found that people imagining their partner sharing a meal with a potential rival experienced more jealousy than when imagining their partner in a face-to-face interaction with a rival without a meal. Sobal et al. (2002) found that shared meals are an important aspect of courtship behavior, with people eating together more frequently as commitment increased. Sharing meals with relatives was found to be the primary way to become acquainted with one another’s family – and served as a signal that the relationship was becoming more serious – while dating mostly revolved around eating together at restaurants or at one another’s home. Taken together, these findings underline that people perceive food sharing as an important indicator of – and means to establish and increase – intimacy, friendship and love.

## REGULATING EMOTIONS WITH COMFORT FOOD

Troisi and Gabriel (2011) posed that the social aspect of eating is an important reason for the emotion regulatory capacity of food. In order to provide infants with safe and secure care, infants’ needs for food, warmth, sleep, soothing, and so forth, need to be regulated in a responsive, sensitive, manner (Gunnar, 1998). Accordingly, children come to associate having their needs met (i.e., feeling warm or satiated) with social cues related to the presence and care of other people (e.g., Williams and Bargh, 2008; IJzerman and Semin, 2009). Emotions and expectations later in life are linked to these childhood regulatory interactions (Hofer, 2006). In rat pups, for instance, the mother’s licking, nursing and grooming behaviors are important factors in the development of the pups’ stress response system (Meaney and Szyf, 2005). When the mother is separated from the pup, the pup’s HPA axis is no longer regulated through nursing and the provision of maternal warmth. The mother’s absence can result in permanent changes of the HPA axis, which, in the long-run, may lead to an increased vulnerability to disease (O’Mahony et al., 2009). In humans, too, early life regulatory interactions are related to the development of the stress response system (e.g., Tarullo and Gunnar, 2006). Moreover, Hofer (2006) posed that through conditioning processes and the establishment of mental schemas and representations, the physiological effects of parent–child regulatory interactions become associated with psychological concepts related to close relationships.

In line with the idea that physiological and psychological processes are related, the literature on embodied cognition highlights the interplay between the body, the environment, and the mind. According to Barsalou (1999) experiences are fixed within a situation, such that contextual information is coded in association with perception (sensory information), action (information about spatial properties and movement) and introspection (mental states, affects, and motivation). Using and combining the information from different modalities can facilitate learning and aid in the representation of abstract concepts (Schubert, 2005). For instance, children experience physical warmth when someone takes care of them, which over time creates an interrelationship between social proximity and physical warmth (Williams and Bargh, 2008; IJzerman and Semin, 2009). The ability to associate the grounded experience of physical warmth with the abstract concept of friendship facilitates knowledge representation. Accordingly, Zhong and Leonardelli (2008) found that when people are subjected to social exclusion and rejection, they feel cold and crave warm food, presumably as a means to compensate for the lack of social proximity (Bargh and Shalev, 2012). Similarly, the grounded experience of tasting something bitter (physical disgust) has been shown to elicit feelings of moral disgust (Eskine et al., 2011), while tasting something sweet increased agreeableness and prosocial behavior (Meier et al., 2012). These findings support the idea that physical experiences such as food offering and sharing can activate related higher-order, more abstract concepts associated with (close) relationships.

Embodied cognition may be particularly relevant in relation to comfort food – “a specific food consumed during a specific situation to obtain psychological comfort” (Wansink and Sangerman, 2000; p. 66). Troisi and Gabriel (2011) reasoned that the appeal of eating comfort food may arise from its association with social proximity, due to a history of frequently consuming these food items in the presence of close relationship partners. Indeed, the researchers found that relationship-related constructs were activated among participants consuming comfort food (chicken soup), but not among participants who did not eat anything. Interestingly, they also found that the effects of a belongingness threat on loneliness was attenuated when participants were instructed to write about the experience of eating a food item that they viewed as comfort food. The authors concluded that “the emotional power of comfort food comes from its connection with relationships and is realized in its propensity to reduce feelings of loneliness” (p. 751). However, for participants with an insecure attachment style comfort food did not buffer loneliness, supposedly because caretaker–child interactions did not allow for the formation of positive mental representations of interpersonal closeness through food intake.

Lupton (1994) expounded that the emotional effect of food lies imbedded in memory, whether these are memories of the social circumstances in which food was consumed, or memories of the soothing and familiar ties to one’s class, ethnic, or religious group that particular food items can come to represent. In fact, food fulfills a comforting role even in highly distressing situations where food ceases to have any nutritional value. In the US, for example, it is customary to allow inmates on death row to order a last meal. An analysis of 193 last meals showed a preference for meals extremely high in fat and carbohydrates (2756 calories on average) and a propensity to ask for food that was familiar, such as specific brands and foods typical for the Southern regions of the United Sates, such as fried food, coleslaw, and pie. The authors suggested – in line with Zhong and Leonardelli (2008) – that especially in such an extreme setting of societal ostracism, selecting food items that are tied to one’s community may provide a source of comfort and, albeit symbolically, social connection (Wansink et al., 2012).

Locher et al. (2005) designed an in-depth study to examine how food preferences are shaped by interpersonal relationships. They asked students to bring their favorite foods to class – food that made them feel good or provided solace. Students were also requested to explain why they had selected these foods. The authors identified four categories of comfort food. *Nostalgic food* reinforced cultural and familial bonds. Participants noted that especially when separated from friends and family, the consumption of nostalgic food (e.g., chicken wings associated with family gatherings), supported their sense of self-identity and the notion that they were part of a social group. *Indulgence food* consisted of luxury food (e.g., sushi) and/or food expansive in calories, fat, or sugar (e.g., cheesecake). *Convenience food* served instant gratification of needs (e.g., potato chips or frozen pizza). Finally, *physical comfort foods* were described as being comforting in texture or temperature (e.g., spun sugar or hot soup).

Additionally comfort food had specific features. First, it elicited a sense of familiarity. Second, comfort food was often reserved for specific situations (e.g., feeling sad or stressed). Third, and most importantly, although comfort food was associated with positive social interactions from the past, students reported consuming it when being alone. These results are in line with Troisi and Gabriel’s (2011) finding that comfort foods were “favorite food, a family tradition, a cultural tradition, something eaten for a holiday, something eaten for a significant family event, a part of the participant’s past, or a reminder of home” (p. 750). Food that was offered in a positive, interpersonal context likely activates the contextual positive emotions and feelings of belongingness upon consumption later in life.

## EMPATHIC EMOTION REGULATION

**FIGURE 1:**
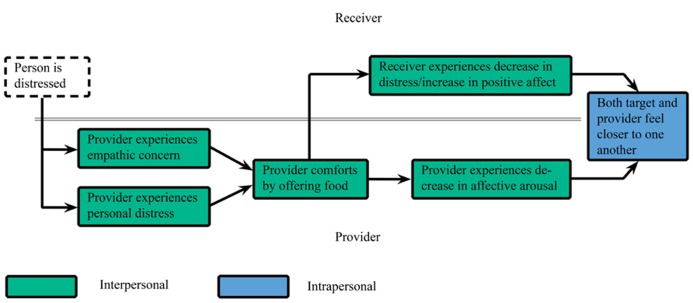
**Example of empathic emotion regulation by food offering**.

Food appears to be an effective means of intrapersonal emotion regulation due to its physiological and psychological properties. It is surprising however, that although the regulatory effects of food originate from interpersonal interactions, this social component has, to our knowledge, received little scientific attention. If people use food to regulate their own emotions, emotion regulation might also underlie people’s offer of food to others. We propose that one mechanism responsible for food offering is EER. EER can be considered an interpersonal regulation system in which an empathic response to another person’s emotional state not only aims to regulate emotion within the provider but across interaction partners as well. **Figure 1** gives an example of this process. EER through food offering can reinforce itself; whenever a food item is successfully used as a regulatory tool, this may increase the association between the food and more positive affect and less negative affect. Therefore, the likelihood that one will use the food to regulate emotions in other social contexts increases.

People are motivated to regulate their own emotions, but empathy – the capacity to understand and react correspondingly to the emotions of others (e.g., Coke et al., 1978) – triggers the motivation to regulate others’ emotions as well. The literature suggests that empathy entails both cognitive and affective responses (Davis, 1994). Perspective taking – trying to understand the emotional state of another person – leads people to sympathize, show concern, and feel compassion for the person in need. These feelings of empathic concern may, in turn, result in a motivation to show support and helping behavior for other-oriented reasons (e.g., compassion, pity). Yet, imagining to be in the position of a person in distress (imaging how one would feel in this situation, versus how the other person feels), may lead to discomfort and personal distress. People are then motivated to attenuate their own arousal, which may trigger helping for more self-oriented reasons (e.g., hedonic avoidance of negative feelings; Coke et al., 1978; Davis, 1994; Decety and Jackson, 2004; Lamm et al., 2007). Davis (1994) suggested that empathic concern and personal distress are not mutually exclusive, and that both may elicit support behavior. Helping and other support behaviors diminish distress in the receiver of support; once the support provider is no longer confronted with a person in distress, the related personal discomfort and arousal will also decrease. Support behavior thus regulates the emotion of the person in distress as well as the arousal-by-proxy brought on by empathic concern.

For example, witnessing a partner’s disappointment of not getting a long sought-after promotion, may trigger feelings of both personal distress and empathic concern, which may motivate EER through food offering of cookies. As a consequence, the partner’s negative affect may lessen and positive affect may increase, due to a combination of physiological effects (Markus et al., 1998; Oliver and Wardle, 1999; Oliver et al., 2000; Dallman et al., 2003) and positive past associations with cookies (Locher et al., 2005; Troisi and Gabriel, 2011). In turn, the provider’s distress and arousal will also decrease. Finally, past associations that exist both within the provider and recipient with cookies and social relationships (e.g., baking cookies with a parent, eating them at parties, sharing them with friends), together with having provided/received support that led both partners to feel better may result in feelings of increased interpersonal closeness.

## EER THROUGH FOOD OFFERING

Of course, there are many different ways to offer support to others, such as giving encouragement, offering advice, helping with a task, or expressing empathy or concern, either verbally or physically through a touch or hug (e. g., Cohen, 2004). However, we propose that EER through food offering is distinct from other support behaviors for several reasons. First, food offering is one of the earliest biobehavioral regulatory interactions between parent and child (Hofer, 2006). As the anthropologist Kathleen Barlow wrote: “if food and feeding are not intrinsic to mothering, they must be nearly so” (2010, p. 339). Inevitably, children form associations between food, emotion regulation, and social proximity.

Second, support behavior has a positive effect on interpersonal relationships, and increases closeness between relationship partners (Devoldre et al., 2010). When food is offered as a support behavior this resonates the associations between food and its social and emotional properties that have developed throughout the lifespan. Therefore, closeness between the provider of food and the recipient should increase both due to the offer of food as a support behavior and due to the feelings of closeness and belongingness that the item of food may already represent for both provider and recipient.

Third, in contrast to other means of EER, food offering is a direct and visceral way to satisfy a basic need in another person while conveying a myriad of social meanings. Because offered food is ingested, its effect entails emotional, psychological, and physiological properties (Locher et al., 2005). Furthermore, from an evolutionary perspective, taking away some of one’s own food resources to feed another person sends a strong, yet implicit message of wanting the other person to live – after all, food is necessary to survive. Well-fed people may not consciously think about how an offer of food will help them survive, but the link between food and survival remains implicitly present through a shared evolutionary past (Bargh et al., 2001; Floyd and Morr, 2003; Parker et al., 2006).

Finally, food offering also has a universal quality. Most other support behaviors are only appropriate in the context of an intimate relationship (e.g., stroking someone’s hair, a hug, a massage) and vary considerably as a function of culture, age, or sex. Food offering can be used as a strategy for EER in any type of relationship. Offering food even to strangers can be appropriate, and it may represent a strategy to establish initial contact as well as to strengthen bonds in already close relationships (Jaeggi and Van Schaik, 2011). Furthermore, food offering can serve to turn enemies into allies. The ability to regulate the emotions of a stranger through food offering may determine the difference between a potential enemy and a potential ally. History is filled with examples where food offering and a shared meal are used as a means for appeasement. For example, the banquet hosted by the Chinese Prime Minister Chou En-lai in 1972 marked the first step in a better relationship between the US and China during the Cold War.

### EER THROUGH FOOD OFFERING – FUNCTIONAL OUTCOMES

The prevalence of food offering to relatives, friends, acquaintances, and even strangers suggests that food offering is exceptionally functional in EER. Support provision can be instrumental, emotional, or informational in nature (e.g., Cohen, 2004). Instrumental support refers to material aid, such as financial support or assistance. Emotional support can consist of expressing empathy and reassurance. Giving guidance or advice to help someone cope with a difficult situation falls under informational support.

How does food offering relate to other forms of support? Witnessing a friend distressed over a break-up or a spouse experiencing work stress is likely to elicit empathic concern and personal distress. We propose that offering chocolate cake to the friend or cooking lasagna for the spouse decreases stress for the recipient and empathic distress for the provider, and strengthens the social bond. However, food offering does not necessarily occur separately or as a replacement of other support behaviors. In fact, food offering can work as a facilitator by creating a setting through which other forms of support can be offered. For instance, in the Jewish tradition the community feeds a person in mourning for seven days following a death (Wolfson, 2005). The offer of food is a way to show caring and support, but it also gives people a reason to visit the house, to talk about the deceased, to notice what other forms of help may be needed, and to check up on the person in mourning.

Furthermore, when people are distraught or unable to talk about what upsets them, the offer of food may allow them to sit down and open up. Food can aid in decreasing overpowering emotions and self-awareness by directing attention to the immediate environment (Heatherton and Baumeister, 1991). Being offered milk and chocolate chip cookies after a long school day may somewhat reduce the threat of disclosing a bad grade among children, or may make it easier to talk about having been bullied. Consequently, the milk and cookies coax a child not to isolate themselves in their room, but instead to sit down and talk about what happened. Food offering may therefore be a support behavior in and of itself, but can also serve as a facilitator of other forms of support.

### EER THROUGH FOOD OFFERING – DYSFUNCTIONAL OUTCOMES

Food offering may become such an effective strategy of EER among some individuals or in some relationships that it may come to replace other forms of support behavior. Accordingly, EER through food offering may play a pivotal role in the development of dysfunctional eating habits and potential weight problems.

Not everyone reacts to stress and negative affect by increasing food intake. An overview by Macht (2008) showed that food intake could also remain unaffected or decrease as a response to intense emotions or stress. Whether food is used as a coping mechanism seems to be predicted primarily by the extent to which people eat as a response to emotional arousal and anxiety rather than hunger (O’Connor et al., 2008). Emotional eating, in turn, has been linked to overweight and eating disorders (Arnow et al., 1995). Parents high in emotional eating may be more likely to (over) employ food offering as EER with their children than parents low in emotional eating (Wardle et al., 2002). If instrumental or informational support – assisting with homework, calling an unfair teacher, talking through a bad day – seem too effortful or has frequently proven to be ineffective, the use of food as a form of emotional support among parents high on emotional eating may be overly tempting. If the physiological and psychological properties of EER through food offering consistently lead to increased positive affect for both receiver and provider, parents may come to be overly reliant on food offering as EER. Nevertheless, effective long-term emotion regulation and the development of constructive coping strategies requires an environment that employs and stimulates a range of support behaviors and various coping strategies (e.g., Landry et al., 2006). Through modeling, parents employing food offering and emotional eating as a substitute for other support behavior may stimulate children to use food as a means to regulate emotion (Snoek et al., 2007).

Providing others with effective support is costly and not always easy, especially in stressful circumstances (Rafaeli and Gleason, 2009). Parents who are too busy, exhausted, or depleted from coping with daily stressors may use food in instances where punishment, minimizing the importance of the emotion, or problem-solving might be more functional and conducive to children’s psychosocial development. Children whose parents over-employ food offering may therefore perceive a lack in social support, which, in turn, has also been shown to lead to emotional eating (Raspopow et al., 2013). Therefore, the literature suggests different pathways through which emotional eating habits can develop. There appears to be a direct pathway in which parents over-employ food as emotion regulation and model this behavior to their children (Snoek et al., 2007), whereas lack of parental social support can indirectly lead children to use food as a means of compensation (Raspopow et al., 2013).

Emotional eating is linked with emotion-oriented coping and avoidance distraction (Raspopow et al., 2013). Giving candy to a crying child may soothe the child faster than figuring out why it is crying. Yet, giving candy could have poor long-term consequences if this strategy becomes habitual, because any underlying problems fail to be addressed and solved. Consistent with these suggestions, a qualitative study of the role of food in families of obese adolescents showed that once food offering has become the primary means of EER, parents find it difficult not to supply food, because they fear this will disrupt their bonding with and influence over the child (Lachal et al., 2012).

Another factor that may lead people to use food offering as a substitute for other support behavior is people’s (in) ability to recognize emotions. Rommel et al. (2012) found that obese participants were less able to identify their own emotions and the emotional states of others than participants of normal weight. Obese participants were also more likely to be emotional eaters, although decreased emotional awareness did not lead to increased emotional eating. In line with previous research (Spoor et al., 2007; Whiteside et al., 2007; Raspopow et al., 2013), the authors suggested that people who are obese and show decreased emotional awareness rely more on emotional eating as a means to regulate emotion due to a lack of alternative strategies. An important question for future research is whether having been raised in an environment where EER through food offering takes precedence over alternative strategies, leads to a restricted range in coping behavior and decreased emotional awareness of self and others.

Parents high in emotional eating and low in awareness of their own and others’ emotional states may be more prone to use food offering as EER than parents who are high in awareness of emotional states. A lack of awareness of the emotional needs of others may lead people to judge EER through food offering as an appropriate response to the distress of others. Additionally, reduced emotional awareness could make it harder to empathize with another person. Devoldre et al. (2010) suggested that personal distress and discomfort (a focus on the self versus the other) leads to less constructive support behavior. Using EER through food offering as a substitute for other, more instrumental, support behavior may reflect a desire to avoid or quickly extinguish personal distress at the expense of more constructive – but perhaps also more confrontational – alternative strategies for support provision. For example, treating a disgruntled spouse to a fancy dinner after weeks of staying late at the office, may seem like an easier fix than reducing one’s work hours.

Finally, it is quite possible that EER through food offering can be a functional mechanism in most environments, but may become problematic in an environment where few alternative strategies for EER are available (e.g., a single mother holding two jobs to make ends meet), that is characterized by a high amount of stressors (e.g., living in a neighborhood with a high crime rate), or where there is easy access to junk foods and convenience foods (e.g., many U.S. college campuses). Low economic status has been shown to be an important predictor of obesity in highly developed countries (McLaren, 2007), possibly because of a higher need for EER and a restricted range of alternative strategies. Furthermore, production companies are eager to highlight the nostalgic, indulgent, convenient, or physical comforting properties of food items as a marketing strategy (Locher et al., 2005). The advertisements tap into pre-existing associations between food and providing or receiving comfort, thereby tempting people to choose instant gratification over alternative strategies of emotion regulation.

## FUTURE DIRECTIONS

The offer of food in early childhood, school, social groups, and close relationships lays the groundwork for the associations between food and emotion, which may become firmly rooted in people’s mental representations and may be passed onto others. So far, research has mainly focused on the intrapersonal antecedents and consequences of using food for emotion regulation. However, a focus on the social processes surrounding food and emotion regulation will aid in increasing societal awareness of food offering as a tool for EER. Greater understanding of the social processes associated with food and their link with emotion regulation may also help in changing the environments in which food offering has become problematic.

Future studies are needed to examine the role of comfort food in EER. We predict that more comfort-type food should be offered when the situation calls for emotion regulation, so as a response to a negative, but not to a neutral emotional state of another person. After consumption of food used as EER, we expect negative affect to decrease in the receiver and distress and empathic concern to decrease in the provider. We also predict that both interaction partners, whether friends or strangers, will feel closer to one another after the food exchange has taken place. Diary studies could be used to examine how EER through food offering varies as a function of daily affect and emotions in relationships and families. Further research is needed to systematically examine the (in) dependence of food offering and other types of support in daily life. We predict that food offering is frequently used to attenuate the daily hassles and stress of other people in the household.

Researchers should differentiate between the direct (physiological) and indirect (social) effects of food offering. Comfort food is often considered to be food with high levels of sugar, fat, or carbohydrates (Wansink et al., 2003). The direct effect these compounds have on neurotransmitters and endocrine responses (Markus et al., 1998; Dallman et al., 2003) should be taken into account when examining the effect of social relationships on the link between food offering and mood.

Possibly, EER through food offering is not merely used to regulate negative emotions, but positive emotions as well. Restrained eaters, for instance, increase food intake after both positive and negative emotions (Cools et al., 1992). Macht et al. (2002) found that men ate more chocolate, and enjoyed eating chocolate more, after an induced positive versus negative mood state. Especially in social settings – where positive affect is often already high – food intake is higher than when eating alone (De Castro, 1994). Research on capitalization suggests that relationship wellbeing is enhanced when others respond actively and constructively to a person sharing good news (Gable et al., 2004). Offering food as a response to capitalization attempts could be a non-verbal signal of shared enthusiasm, motivated by perspective taking. Investigating eating behavior from a social perspective will contribute to enhance our understanding of the psychosocial and emotional factors that put people at risk for dysfunctional patterns of EER through food offering.

## CONCLUSION

Given the dramatic weight gain and rise in obesity in countries all over the world (Wang and Lobstein, 2006), the scientific study of food has become increasingly relevant. We strongly urge that research in this domain should include the systematic examination of the social aspects and interpersonal functions of eating. Food preferences and eating behavior are shaped in childhood and develop under the influence of relatives, peers, partners, and the socio-cultural environment. The influence of social norms regarding eating behavior remains strongly present in adulthood. Many social interactions revolve around shared meals, and even when a meal is consumed alone food items can elicit a sense of belonging through associations and memories.

In the developed world, overweight has become a more pressing problem than malnutrition. People overconsume and use food not merely to satisfy hunger, but also hedonically and as a response to emotional states. We proposed that food offering may be part of EER in relationships: being exposed to another person’s negative emotions, and potentially positive emotions as well, elicits empathic concern (Coke et al., 1978), leading people to use food to regulate not merely their own, but also others’ emotions. Food offering thus provides a way of coping with distress and empathic concern, as well as an effective means of offering social support, resulting in increased positive affect across interaction partners and an increase in interpersonal closeness.

EER through food offering, whether as a response to positive or negative emotions, may be adaptive, but can lead to dysfunctional patterns when over-employed. Knowledge of which individual characteristics, social experiences, and external factors, such as stress, may exacerbate food offering as a means to regulate emotion – at the expense of other support behaviors – could contribute to the development of interventions for families struggling with overweight and obesity. Determining the role of EER through food offering would be an important step in research focused on social support, coping mechanisms, and eating behavior.

## Conflict of Interest Statement

The authors declare that the research was conducted in the absence of any commercial or financial relationships that could be construed as a potential conflict of interest.
